# Correction: Assani et al. Beyond HOMA-IR: Comparative Evaluation of Insulin Resistance and Anthropometric Indices Across Prediabetes and Type 2 Diabetes Mellitus in Metabolic Syndrome Patients. *Life* 2025, *15*, 1845

**DOI:** 10.3390/life16020333

**Published:** 2026-02-14

**Authors:** Mohamed-Zakaria Assani, Lidia Boldeanu, Anda Lorena Dijmărescu, Daniel Cosmin Caragea, Ionela Mihaela Vladu, Diana Clenciu, Adina Mitrea, Alexandra-Ștefania Stroe-Ionescu, Mariana-Emilia Caragea, Isabela Siloși, Mihail Virgil Boldeanu

**Affiliations:** 1 Doctoral School, University of Medicine and Pharmacy of Craiova, 200349 Craiova, Romania; mohamed.assani@umfcv.ro (M.-Z.A.); alexandra.stroe@yahoo.com (A.-Ș.S.-I.); mariana.emilia77@yahoo.com (M.-E.C.); 2 Department of Immunology, Faculty of Medicine, University of Medicine and Pharmacy of Craiova, 200349 Craiova, Romania; isabela_silosi@yahoo.com (I.S.); mihail.boldeanu@umfcv.ro (M.V.B.); 3 Department of Microbiology, Faculty of Medicine, University of Medicine and Pharmacy of Craiova, 200349 Craiova, Romania; lidia.boldeanu@umfcv.ro; 4 Department of Obstetrics and Gynecology, Faculty of Medicine, University of Medicine and Pharmacy of Craiova, 200349 Craiova, Romania; 5 Department of Nephrology, Faculty of Medicine, University of Medicine and Pharmacy of Craiova, 200349 Craiova, Romania; 6 Department of Diabetes, Nutrition and Metabolic Diseases, Faculty of Medicine, University of Medicine and Pharmacy of Craiova, 200349 Craiova, Romania; ionela.vladu@umfcv.ro (I.M.V.); dianaclenciu@yahoo.com (D.C.); ada_mitrea@yahoo.com (A.M.)

## Figure Correction

In the original publication [1], there is an error about Figure 2. The corrected Figure 2 appears below. The authors and the Editorial Office confirm that these changes do not affect the scientific conclusions of the study. This correction was approved by the Academic Editor. The original publication has also been updated.

## Figures and Tables

**Figure 2:**
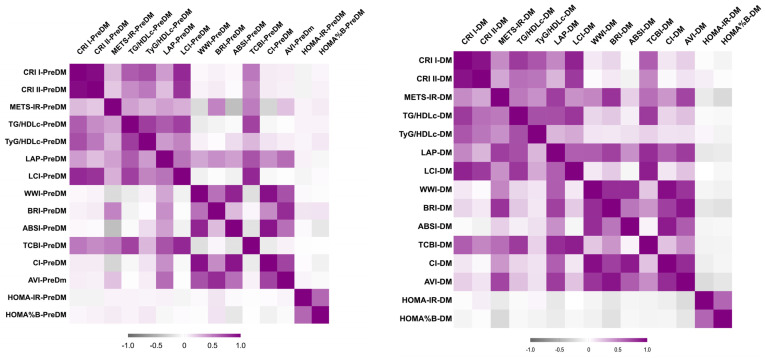
Correlation Heatmap of PreDM cohort (**left**) and T2DM cohort (**right**).
